# Draft Genome Sequence of a Propanotroph, Rhodococcus sp. Strain ENV425, Capable of Degrading Methyl *tert*-Butyl Ether and *N*-Nitrosodimethylamine

**DOI:** 10.1128/genomeA.00051-18

**Published:** 2018-02-22

**Authors:** Peter R. Tupa, Hisako Masuda

**Affiliations:** a Department of Mathematics, Indiana University Kokomo, Kokomo, Indiana, USA; b Department of Chemistry, Indiana University Kokomo, Kokomo, Indiana, USA

## Abstract

In this study, the draft genome of Rhodococcus sp. strain ENV425 was determined. The propane-grown strain ENV425 cometabolically degrades environmental contaminants such as methyl *tert*-butyl ether and *N*-nitrosodimethylamine. The sequence revealed the presence of multiple hydrocarbon metabolic genes that could play pivotal roles in the biodegradation of pollutants.

## GENOME ANNOUNCEMENT

Propane is a gaseous three-carbon alkane. Bacteria capable of growing on propane as a sole source of carbon, called propanotrophs, have been isolated from various environmental samples (1–3). Multiple oxygenases were shown to catalyze the initial oxidation of propane, including soluble methane monooxygenase (sMMO), propane monooxygenase (PMO), particulate methane monooxygenase, engineered alkane hydroxylase (AlkB), and the CYP153 family of cytochrome P450 (2, 4–8). In propane-grown cells, multiple oxygenases are often simultaneously expressed (2, 3, 9).

Interestingly, a variety of propane oxygenase homologues and propane-inducible oxygenases have flexible substrate ranges and are capable of oxidizing environmental contaminants such as 1,4-dioxane and *N*-nitrosodimethylamine (NDMA) (6, 10, 11). This allows propane-grown bacteria to degrade non-growth-supporting contaminants via cometabolism (1, 10, 12). The application of cometabolic degradation by propanotrophs for the remediation of contaminated ground water has attracted attention, as the physiochemical separation from water and subsequent degradation of these compounds are costly processes (13).

Rhodococcus sp. strain ENV425 cometabolically degrades methyl *tert*-butyl ether (MTBE) and NDMA (1, 12). The growth of this strain on propane and on a few putative metabolic intermediates of propane metabolism, such as 2-propanol and acetone, has supported the cometabolism of MTBE (1). Our earlier PCR-based screen identified a few genes encoding putative hydrocarbon oxygenases with a propane-inducible expression pattern (12). However, the enzyme(s) responsible for the oxidation of MTBE, as well as propane, remains unknown.

In this study, the draft genome of strain ENV425 was deciphered by next-generation sequencing and *de novo* assembly. Genomic DNA was extracted as previously described (12) and sequenced with an Illumina HiSeq 2000 sequencer, which generated 11,520,830 reads. Reads were assembled using Velvet (14), yielding 751 contigs with an *N*_50_ value of 40,554 bp. The total size of the draft genome was 6,191,379 bp, with 70.3% G+C content. The annotation of the genome by the NCBI Prokaryotic Annotation Pipeline and Rapid Annotations using Subsystems Technology (RAST) server revealed the presence of 5,707 protein-coding sequences, 48 tRNA genes, and 5 rRNA genes (15).

Multiple genes encoding putative hydrocarbon oxygenases were identified. Gene clusters encoding two soluble diiron monooxygenases (SDIMOs), two AlkBs, and one benzene dioxygenase were identified. Both SDIMO α-subunit sequences were nearly identical to the sequences of putative sMMO/PMO from many Rhodococcus strains. The involvement of these oxygenases in the initial step of propane metabolism will be examined in future studies. Whether or not these oxygenases are simultaneously expressed in propane, as well as their physiological roles, will also be investigated. The genome sequence exhibited the highest identity to that of *Mycobacterium* sp. strain MCS. However, the gene content and genome organization lacked obvious conservation among the two genomes, suggesting numerous recent genome rearrangements and/or gene acquisitions via horizontal gene transfer.

### Accession number(s).

The complete genome sequence of Rhodococcus sp. strain ENV425 has been deposited in GenBank under the accession no. PCZU00000000.
